# Identification of Prognosis-related Hub RNA Binding Proteins Function through Regulating Metabolic Processes in Tongue Cancer

**DOI:** 10.7150/jca.52156

**Published:** 2021-02-22

**Authors:** Tao Shen, Meiting Wang, Xiangting Wang

**Affiliations:** 1 Department of Geriatrics, The First Affiliated Hospital of USTC, Division of Life Sciences and Medicine, Hefei National Laboratory for Physical Sciences at the Microscale, University of Science and Technology of China, Hefei, 230026, China.; 2 College of Liren, Yanshan University, Qinhuangdao, 066004, China.; 3 Department of Neurobiology and Biophysics, School of Life Sciences, University of Science and Technology of China, Hefei, 230026, China.

**Keywords:** RNA binding protein, tongue cancer, metabolic process, risk score, prognostic model.

## Abstract

RNA binding proteins (RBPs) are dysregulated and associated with the occurrence and development in various malignant tumors. However, the role of RBPs in tongue cancer are largely unclear. Here, by integrating the differential gene expression analysis and the Weighted Gene Co-expression Network Analysis (WGCNA) of TCGA-retrieved RNA-seq data, we identified a total of 171 differential co-expression RBPs. Then, in a protein-protein interaction (PPI) network containing 134 nodes (RBPs) and 315 network edges (RBP-RBP interacting networks), the top 30 hub RBPs were identified using the CytoHubba plugin of Cytoscape. Furthermore, we investigated the expression and prognostic value of these RBPs and their highly correlated networks. Among them, six RBPs (PGK1, SLC20A1, LEPR, CYP19A1, ZC3H12D, and PFKM) were shown to be the prognosis-related hub RBPs (prhRBPs). Based on these hub RBPs, we constructed a prognostic model and found that the patients in the high-risk group had dramatically poor overall survival compared to those in low-risk group. In addition, we validated the prognostic model in GSE41613, another tongue cancer patient cohort from GEO datasets. The time-dependent receiver operating characteristic (ROC) analysis of the prognostic model further confirmed the predictive capability of the risk model for tongue cancer. As suggested in functional annotation analysis, we found an intensive enrichment of these prhRBPs in metabolic pathways, including AMPK, HIF-1 signaling pathway, Glycolysis, and steroid hormone biosynthesis. Together, our study revealed the underlying role of RBP in tongue cancer biology and potentially unveiled novel targets for cancer therapy.

## Introduction

Tongue cancer is the most frequent type of malignancy in the oral cavity, and is characterized by remarkable aggressive biological behavior 1-4. This is partly due to that tongue cancer is comparatively silent and progresses fast from a premalignant state into invasive carcinoma. This feature causes delay in diagnosis and thus leading to poor prognosis 5, 6. Thus, even the currently understanding of cancer development and progression is rapidly increasing, tongue cancer is still a serious health issue in many counties with significantly lower 5-year survival rate in patients 7, 8. Hence, expanding our understanding of the pathophysiological mechanism to develop effective methods for early screening and diagnosis and identification of new therapeutic targets are urgently required for tongue cancer.

RNA binding proteins (RBPs) are a class of proteins that interacting with a variety type of RNAs. To date, more than 3800 human RBPs were determined in diverse cell types 9-12. These RBPs establish highly dynamic interactions with other proteins, coding and/or noncoding RNAs, and influence a variety of physiological and pathological processes including cancer 13. Several investigations have shown that RBPs are dysregulated in different cancer types, and can impact on the expression and function of oncoproteins and tumor-suppressor proteins 14-23. However, it remains largely unclear of the role of RBPs in tongue cancer. Thus, global analysis of the function impact of RBPs will provide better understanding of tongue cancer and new insight into tongue cancer therapy.

In the present study, we downloaded the RNA-seq and clinical datasets from TCGA and GEO. By utilizing differential expression analysis and WGCNA, we constructed the link between the expression and tongue cancer patients' clinical features. GO and KEGG analyses were carried out to reveal the underlying functional mechanisms in metabolic processes of RBPs in tongue cancer. Moreover, by unveiling a number of tongue cancer prognosis-related hub RBPs, our results may shed new light on understanding RBPs-directed network underlying tongue cancer progression. These hub RBPs may provide potential biomarkers for diagnosis and prognosis.

## Materials and methods

**Figure 1:**
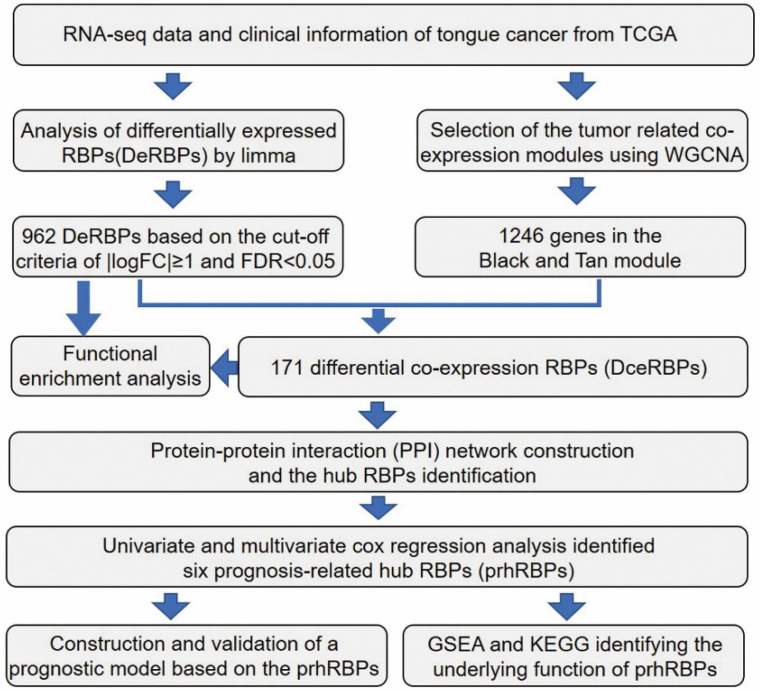
Schematic view of the procedures for analyzing RBPs in tongue cancer.

The workflow of the hub RBPs analysis pipeline is shown in Figure 1.

We elaborate on each step in the following sub-sections.

### Datasets from TCGA and GEO database

Gene expression profiles and corresponding clinical information of tongue cancer were freely downloaded from TCGA (https://portal.gdc.cancer.gov/) and GEO (https://www.ncbi.nlm.nih.gov/gds). To perform an unbiased analysis, all the tongue cancer patients (147 tumor tissues and 15 normal tissues) which could be searched from TCGA database with the information of gene expression, and clinical data were included. A total of 56753 genes (Ensemble ID) were transferred to gene symbols. The data had been generated by using the Illumina HiSeq 2,000 platform, and were annotated to a reference transcript set of human hg38 gene standard track. In addition, normalized expression profiles of GSE41613, another gene expression file from 97 tongue cancer patients were obtained from the GEO database. GSE41613 contains completed clinical information for the prognosis-related dysregulated gene analyses (Table S7). GSE41613 was chosen as a validation cohort because GSE41613 provided an independent group of patients compared with TCGA. GSE41613 consisted of 97 tumor samples, which were studies with the GPL570 [HG-U133_Plus_2] Affymetrix Human Genome U133 Plus 2.0 Array platform. Probes were converted to the gene symbols based on a manufacturer-provided annotation file and duplicated probes for the same gene were removed by determining the median expression value of all its corresponding probes.

### Data processing

To identify the differently expressed genes (DEGs) between tongue normal and tumor tissue, we used the negative binomial distribution method. The Limma package (http://www.bioconductor.org/packages/release/bioc/html/limma.html) was applied to perform the analysis. The Limma package was based on the negative binomial distribution. It fits a generalized linear model for each gene and uses empirical Bayes shrinkage for dispersion and fold-change estimation. All raw data was preprocessed by Limma package and excluded genes with an average count value less than 1. In addition, we also used Limma package to identify the differently expressed RBPs (DeRBPs) in view of |log2 fold change (FC)|≥1 and false discovery rate (FDR)<0.05.

### Co-expression network analysis

The gene expression data profiles of TCGA were constructed to gene co-expression networks using the *WGCNA* package in R 24. In our study, the most abundant 14334 genes were used in the co-expression network analysis. Low abundance genes were eliminated as their abundance tends to be highly biased. To build a scale-free network, soft powers β = 3 and 20 were selected using the function pickSoftThreshold. Next, the adjacency matrix was created by the following formula: aij = |Sij|β (aij: adjacency matrix between gene i and gene j, Sij: similarity matrix which is done by Pearson correlation of all gene pairs, β: softpower value), and was transformed into a topological overlap matrix (TOM) as well as the corresponding dissimilarity (1-TOM). Afterwards, a hierarchical clustering dendrogram of the 1-TOM matrix was constructed to classify the similar gene expressions into different gene co-expression modules. Modules with high correlation coefficient were considered candidates relevant to clinical traits, and were selected for subsequent analysis.

### GO enrichment and KEGG pathway analysis

The biological functions of DeRBPs and Differential co-expression RBPs (DceRBPs) were comprehensively detected by GO enrichment and kyoto encyclopedia of genes and genomes (KEGG) pathway analysis. The GO analysis terms including cellular component (CC), molecular function (MF), and biological process (BP). All enrichment analyses were carried out by R package *clusterProfiler*, *org.Hs.eg.db*, and *enrichplot* (https://bioconductor.org/packages/clusterProfiler/;https://bioconductor.org/packages/org.Hs.eg.db/; https://bioconductor.org/packages/enrichplot/). Both P and FDR values were less than 0.05 as statistically significant.

### PPI network construction and hub RBP identification

The 171 DceRBPs were submitted to the STRING database 25. Using the STRING database, genes with a score ≥ 0.4 were chosen to build a network model visualized by Cytoscape (v3.7.2) 26. Maximal Clique Centrality (MCC) algorithm was reported to be the most effective method of finding hub nodes in a co-expression network 27. Thus, in this study, the MCC of each node was calculated by CytoHubba, a plugin in Cytoscape. The genes with the top 30 MCC values were considered as hub genes. And the TOP30 hub RBPs and their first stage nodes are used for the following prognostic model construction step.

### Prognostic model construction and validation

Based on the data from the TCGA database, univariate Cox regression analysis was performed on the hub RBPs and their first stage nodes using the *survival* package in R software 28. A log-rank test was executed to screen the significant candidate prhRBPs further. Subsequently, based on the above preliminary screened significant candidate RBPs, we constructed a multivariate Cox proportional hazards regression model and calculated a risk score to assess patient prognosis outcomes. The risk score formula for each sample was as follows:

Risk score = k1× Exp 1+ k2× Exp 2+…+ ki× Exp i

where* k* represents the coefficient value, and *Exp* represented the gene expression level. According to the median risk score survival analysis, tongue cancer patients were divided into low-risk and high-risk groups. A log-rank test compared the difference of overall survival (OS) between the two subgroups. Additionally, a time dependent receiver operating characteristic (ROC) curve analysis was implemented by using the above model 29. Besides, 97 tongue cancer patient samples with reliable prognostic information from the GSE41613 dataset were used as a validation cohort to confirm the predictive capability of this prognostic model.

### Nomogram construction

The clinically-relevant candidate variables (age, gender, tumor stage, and riskscore) were collected from the TCGA and GEO-retrieved tongue cancer patients. Cox regression analysis was performed to calculate the hazard ratios (HRs) and 95% confidence intervals (CIs) of the putative prognosticators. The result shows that only the continuous variable riskscore based on the six hub RBPs were independently associated with overall survival (OS) of TCGA- and GSE41613-retrieved tongue cancer patients (Figure S4). Thus, the nomogram model based on the six hub RBPs was formulated by using rms R package to predict survival of tongue cancer patients 30. Based on the nomogram model, each RBP was ascribed a weighted point. Together, the six hub RBPs were applied for predicted the prognosticated survival.

## Result

### Identification of differently expressed RBPs (DeRBPs) in TCGA-retrieved tongue cancer patients

**Figure 2:**
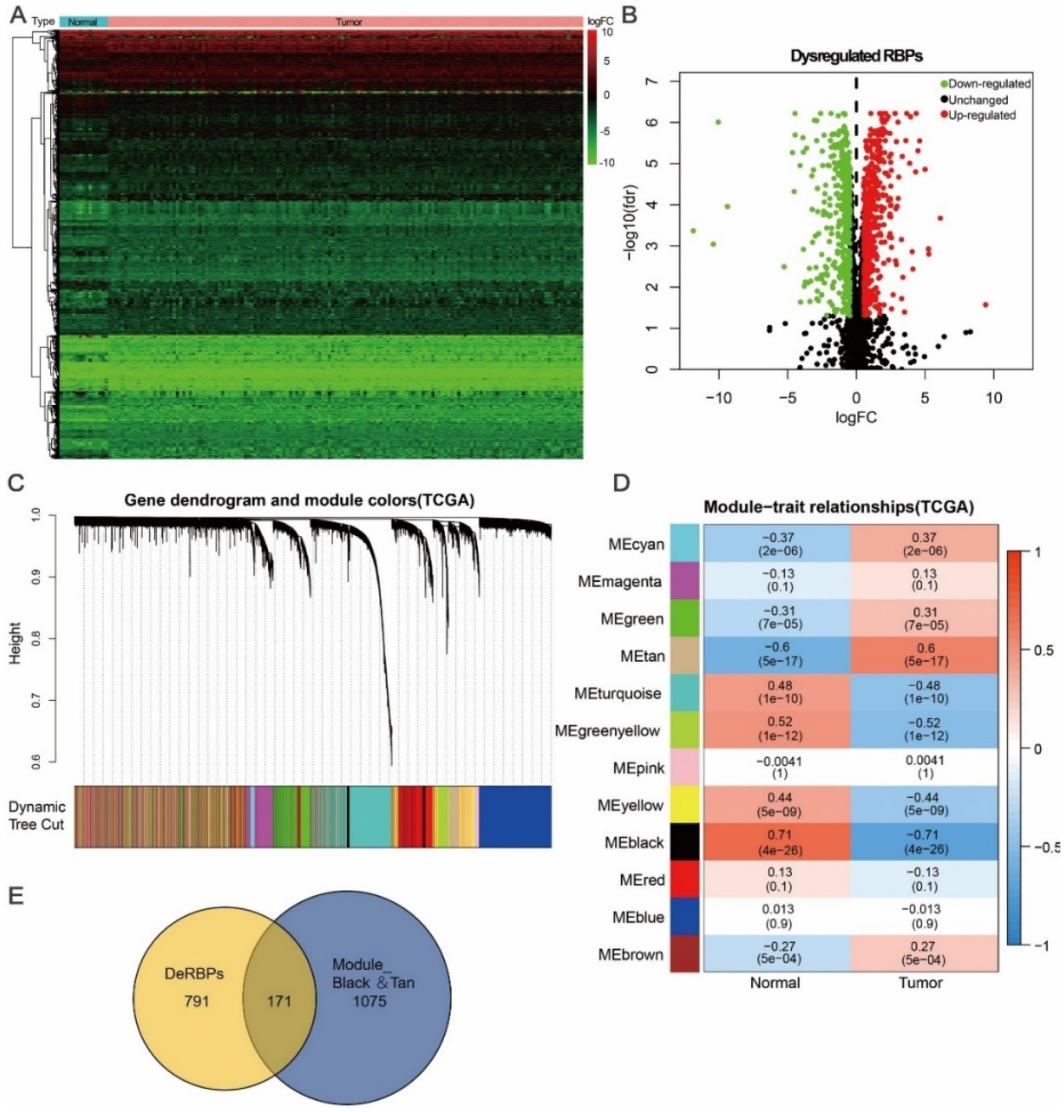
** Identification of differential co-expression RBPs (DceRBPs) in TCGA-retrieved tongue cancer patients.** (A) Heat map of the RBPs in tongue patients. Normal, n=15; Tumor, T=147. (B) Volcano plot of DeRBPs in tongue patients. Down-regulated: Down-regulated RBPs in TCGA-retrieved tongue cancer patients *v.s.* normal patients; Up-regulated: Up-regulated RBPs in TCGA-retrieved tongue cancer patients *v.s.* normal patients; Unchanged: RBPs which shows no expression levels changes in TCGA-retrieved tongue cancer patients *v.s.* normal patients. (C) The Cluster dendrogram of co-expression network modules was ordered by a hierarchical clustering of genes based on the 1-TOM matrix. Each module assigned with different color. (D) Module-trait relationships. Each row corresponds to a color module and column corresponds to a clinical trait (cancer or normal). Each cell contains the corresponding correlation and P-value. (E) Venn diagram of DeRBPs and genes enriched in the highest normal or tumor-trait related modules (Black and Tan).

Here, to investigate the role of RBPs in tongue cancer, we conducted a systematic analysis of key roles and prognostic values of RBPs. The study design was illustrated in Figure 1. We first downloaded the RNA-seq and clinical datasets from TCGA, including 147 tumor tissues and 15 normal tissues. The “Limma” packages were implemented to handle the data and discovered the differently expressed genes. A total of 3823 RBPs were then included in the analysis (Figure S1, Table S1), and 962 RBPs met the screening standard (*P*<0.05, |log2(FC)| >1.0), which consist of 531 up-regulated and 431 down-regulated RBPs (Figure 2A, B, Table S2).

### DeRBPs are mainly enriched in cell progression and metabolic processes

To gain further biological insights into these identified DeRBPs, we uploaded and analyzed the divided up-regulated or down-regulated RBPs. GO analysis mainly enriched the up-regulated RBPs in the biological process (BP) related to cell cycle regulation, nuclear division, and chromosome segregation. (Figure S2A). Through the cellular component (CC) analysis, we found the increased DeRBPs were significantly enriched in focal adhesion, cell-substrate junction, or spindle and chromosomal region (Figure S2A). In terms of molecular function (MF), the increased DeRBPs were notably enriched in cell adhesion molecule binding, ATPase activity, RNA/DNA binding and catalytic activity (Figure S2A). In addition, KEGG analysis enriched the up-regulated RBPs in Regulation of actin cytoskeleton, Cell cycle, virus infection like human papillomavirus (HPV), human immunodeficiency virus (HIV), etc., and cancer metabolic processes including proteoglycans in cancer, HIF1 signaling pathway, Glycolysis/Gluconeogenesis, Central carbon metabolism in cancer, etc. (Figure S2B). For the down-regulated RBPs, we found they were significantly enriched in biological process related to multiple catabolic processes including small molecule catabolic process, RNA catabolic process, etc., and translation initiation (Figure S2C). CC analysis correspondently enriched the decreased DeRBPs in mitochondrial matrix, ribosome, and cytosolic part (Figure S2C) Moreover, through the MF analysis, we found the decreased DeRBPs were enriched in coenzyme binding, structural constituent of ribosome, and translation regulator activity (Figure S2C). KEGG dramatically enriched the down-regulated RBPs in Carbon metabolism, Ribosome, Amino acid (Aa) degradation, TCA cycle, and Fatty acid metabolism and degradation (Figure S2D). In summary, we found the up-regulated RBPs functioned in both cytosolic, nuclear and even extracellular membrane with multiple functions like regulation of cell cycle, cell division, and metabolic processes while the down-regulated RBPs mainly functioned in cytosolic and mitochondrial with the abilities to regulate metabolism.

### Identification of tumor related differential co-expression RBPs (DceRBPs) in TCGA-retrieved tongue cancer patients

Except for differential gene expression analysis, Weighted Gene Co-expression Network Analysis (WGCNA) is another powerful method to study transcriptomics 24. WGCNA can be used to detect co-expression modules of highly correlated genes and interested modules associated with clinical traits providing profound insight into predicting the functions of co-expression genes and finding genes that play key roles in human diseases 31, 32. In order to find the tumor related functional clusters in tongue cancer patients, we constructed the gene co-expression networks from the TCGA datasets with the *WGCNA* package. We totally obtained 12 distinct modules with each module assigned a color (Figure 2C). Genes clustering in each module were shown in Table S3. Next, we plotted a heatmap of module-trait relationships to evaluate the association between each module and two clinical traits (Cancer and Normal) (Figure 2D). The result shown that among the 12 modules, the black and tan modules have the highest association value of either normal tissues or cancer tissues (Trait-Normal: black module: r = 0.71, p = 4e-26; Trait-Cancer: tan module: r = 0.6, p = 5e-17).

In Figure 2A and 2B, a total of 962 DeRBPs were found to be dysregulated in tumor tissues. To further unearth RBPs highly correlated with tongue cancer, we searched genes in both DeRBPs list and the black/tan modules. As shown in Figure 2E, 171 overlapped differential co-expression RBP (DceRBP) genes were identified as tumor or normal highly correlated dysregulated RBPs. As suggested in functional annotation analysis using the R clusterProfiler package, these RBPs were mainly enriched in terms of various metabolic process (BP), focal adhesion and cell-substrate junction (CC), and multiple cell adhesion molecule binding (MF) (Figure S3A). In addition, KEGG analysis dramatically enriched these RBPs into terms of various metabolism related processes (Carbon metabolism, Glycolysis, HIF-1 signaling pathway, Biosynthesis of amino acids, and Valine, leucine and isoleucine degradation), Regulation of actin cytoskeleton, Protein processing in endoplasmic reticulum, Focal adhesion, and Tight junction (Figure S3B).

### PPI network construction and hub RBPs identification

**Figure 3:**
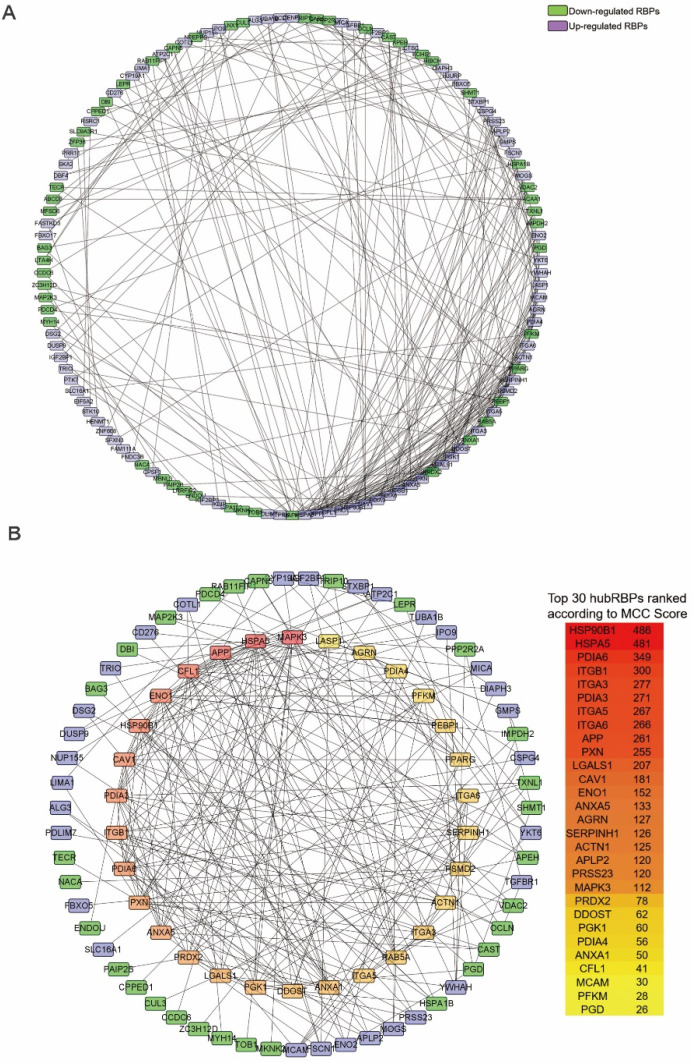
** Identification of the hub RBPs from the constructed DceRBPs' PPI networks.** (A) Protein-protein interaction network of DeRBPs. Edges represent the protein-protein associations. The purple nodes represent up-regulated RBPs, while the green nodes represent down-regulated RBPs. (B) Identification of the hub genes from the PPI network using maximal clique centrality (MCC) algorithm. The red nodes represent RBPs with a high MCC score, while the yellow nodes represent RBPs with a low MCC score.

The PPI network of the DceRBPs was established by using the STRING database, which incorporated 134 nodes and 315 edges (Figure 3A). Then, we searched the hub genes from the PPI network by using the MCC algorithm of CytoHubba plugin (Figure 3B). According to the MCC score, the top 30 highest-scored genes were selected as the hub RBP genes (Table S4).

### Identification of the prognosis-related hub RBPs

**Figure 4:**
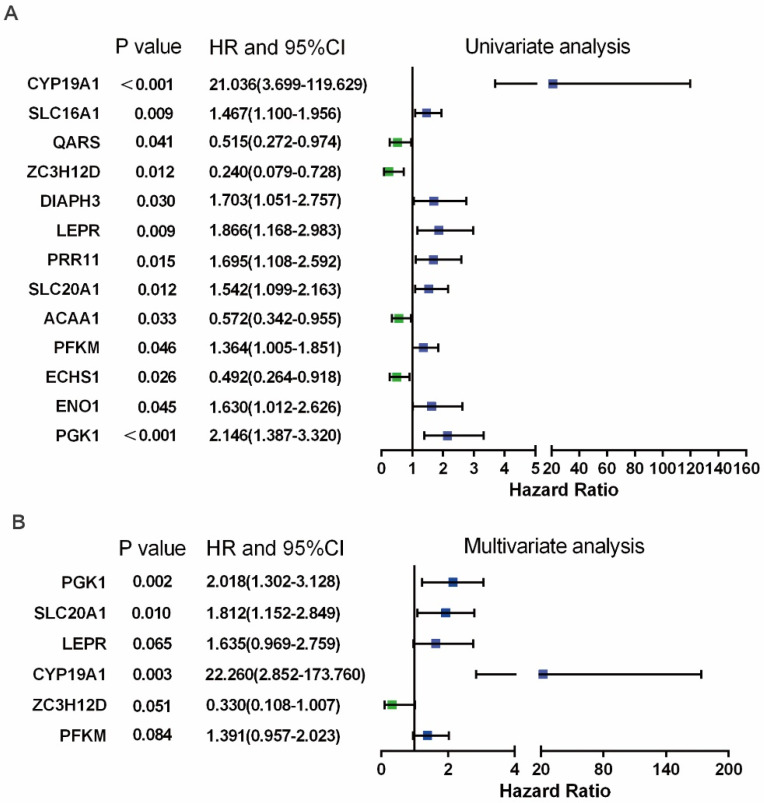
** Prognosis-related hub RBPs (prhRBPs) were identified through univariate and multivariate cox analyses.** (A) Univariate Cox regression analysis to identify the candidate prognosis-related hub RBPs. (B) Multivariate Cox regression analysis to identify prognosis-related hub RBPs.

In Figure 3B, PPI analysis revealed a total of 126 RBPs, including Top30 Hub RBPs and their first stage nodes. To further investigate the prognostic significance of these RBPs, we performed a univariate cox regression analysis and obtain 13 prognosis-associated candidate RBPs (Figure 4A). We subsequently performed the multiple stepwise cox regression analysis to investigate the impact of these 13 prognostic-associated candidate hub RBPs on patient survival time and clinical outcomes. PGK1, SLC20A1, LEPR, CYP19A1, ZC3H12D, and PFKM were found to be independent predictors in tongue cancer (Figure 4B, Table S5).

### Prognosis-related risk model construction and analyzing

Utilizing the aforementioned prognosis-related hub RBPs (PGK1, SLC20A1, LEPR, CYP19A1, ZC3H12D, and PFKM), we constructed a predictive model. The risk score of each patient was calculated according to the following formula:

*Risk Score* = (0.7021×*Exp*PGK1) + (0.5942×*Exp*SLC20A1) + (0.4919×*Exp*LEPR) + (3.1028×*Exp*CYP19A1) + (-1.1087×*Exp*ZC3H12D) + (0.3301×*Exp*PFKM)

**Figure 5:**
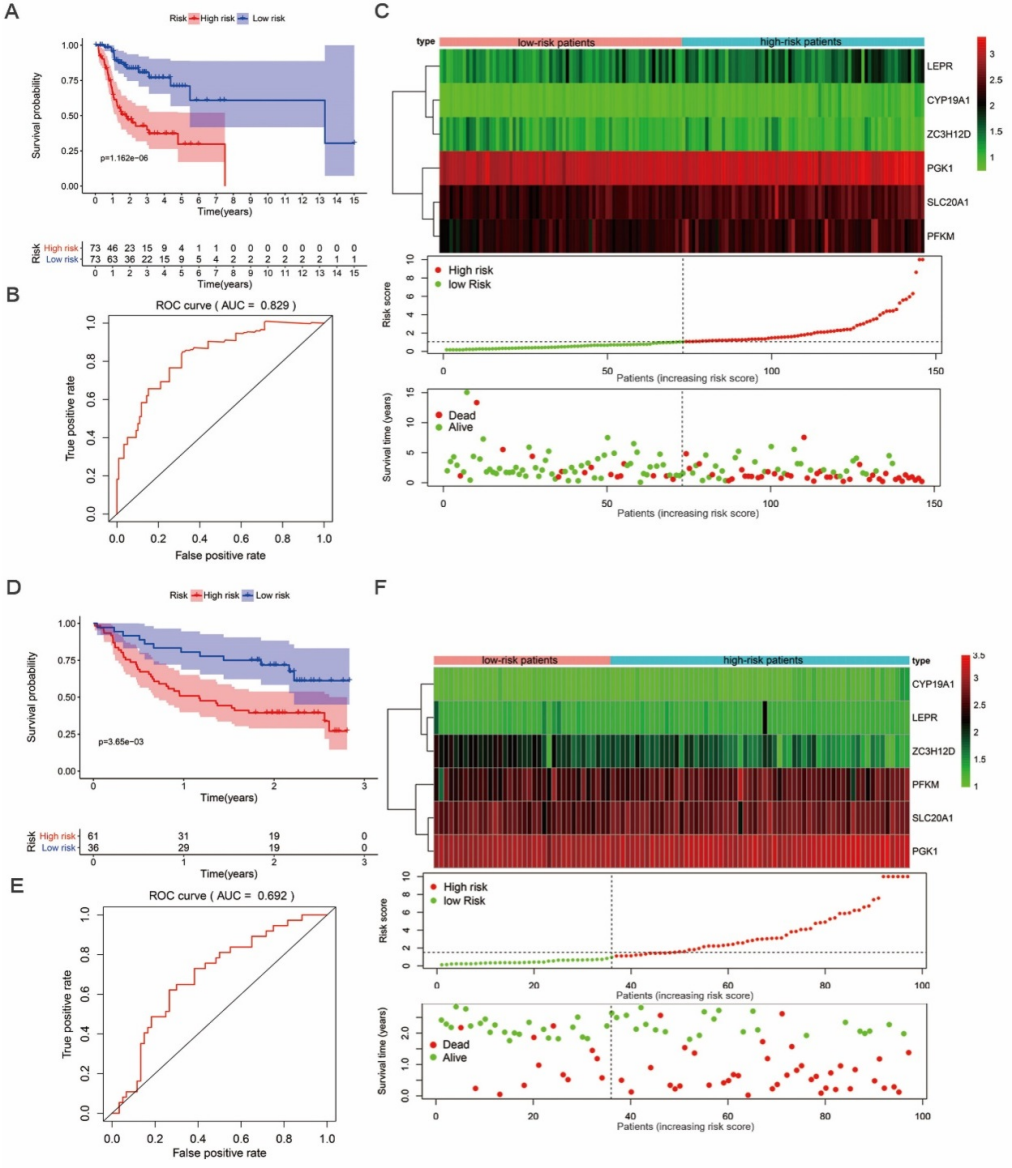
** Survival analysis revealed high-risk score are positively correlated to poor survival.** (A) Survival analysis of low- and high-risk subgroups in the TCGA train group. (B) ROC curves for forecasting overall survival (OS) based on risk score in the TCGA train group. (C) Expression heat map, risk score distribution, and survival status in the TCGA train group. (D) Survival analysis of low- and high-risk subgroups in the GEO test group; (E) ROC curves for forecasting overall survival (OS) based on risk score in the GEO test group. (F) Expression heat map, risk score distribution, and survival status in the GEO test group.

We then proceeded to a survival analysis to assess the predictive ability. 147 tongue cancer patients from TCGA, designated as TCGA train group, were classified as low-risk or high-risk subgroups according to the median risk score. Then, we conducted a survival analysis to assess the predictive ability in TCGA train group. Our results indicated that the patients' overall survival (OS) rate was dramatically lower in the high-risk group compared to patients in low-risk group (Figure 5A). To further evaluate the prognostic ability of the prognosis-related hub RBPs, we executed a time-dependent ROC analysis. As the area under the ROC curve (AUC) of this hub RBPs-based risk model was 0.829, the result indicated the model has a well diagnostic performance (Figure 5B). The expression heatmap of prognosis-related hub RBPs, survival status and risk score of patients were displayed in Figure 5C. Together, these data indicated a predictable power of the prognostic model based on the hub RBPs and risk scores.

In addition, we evaluated whether the hub RBPs-based risk model has similar prognostic value in another tongue cancer patient cohort. We included 97 tongue cancer patients from GSE41613, designated as GEO test group, into the risk score analyses. Similarly, we found that patients with a high-risk score also showed a poor OS than those with a low-risk score in the GEO test group (Figure 5D). In addition, the AUC score of 0.692 also showed a moderate diagnostic performance (Figure 5E). The expression heatmap of hub RBPs, survival status and risk score of patients were displayed in Figure 5F. These results again suggested that the prognostic model derived from the hub RBPs-based risk score has well sensitivity and predictability.

### Validation of the prognostic value of prognosis-related hub RBPs

As the hub RBPs-based risk score has shown the predictive ability in both TCGA and GEO-retrieve tongue patients, we assessed and compared the prognostic significance of different clinical characteristics in tongue patients by performing COX regression analysis. In TCGA datasets, the results showed that tumor grade, stage, and risk score was correlated with OS of tongue cancer (*P*<0.05) in univariate analysis (Figure S4A, left panel). However, only risk score shown to be an independent prognostic factor correlated with OS through multiple regression analysis (*P*<0.05) (Figure S4A, right panel). In GEO datasets, the results showed that only risk score was independent prognostic factors correlated with OS of tongue cancer in both univariate and multivariate analysis (Figure S4B).

In addition, to explore the independent prognostic value of prognosis-related hub RBPs in tongue cancer, we performed the Kaplan Meier-plotter method to determine the relationship between these hub RBPs and OS. A total of four of the six hub RBPs (PGK1, CYP19A1, PFKM, and ZC3H12D) were identified by Kaplan Meier-plotter server. The results of log-rank test demonstrated that PGK1, CYP19A1, PFKM, and ZC3H12D were associated with the OS in tongue cancer patients (Figure S5).

### Construction of a nomogram based on the prognosis-related hub RBPs

**Figure 6:**
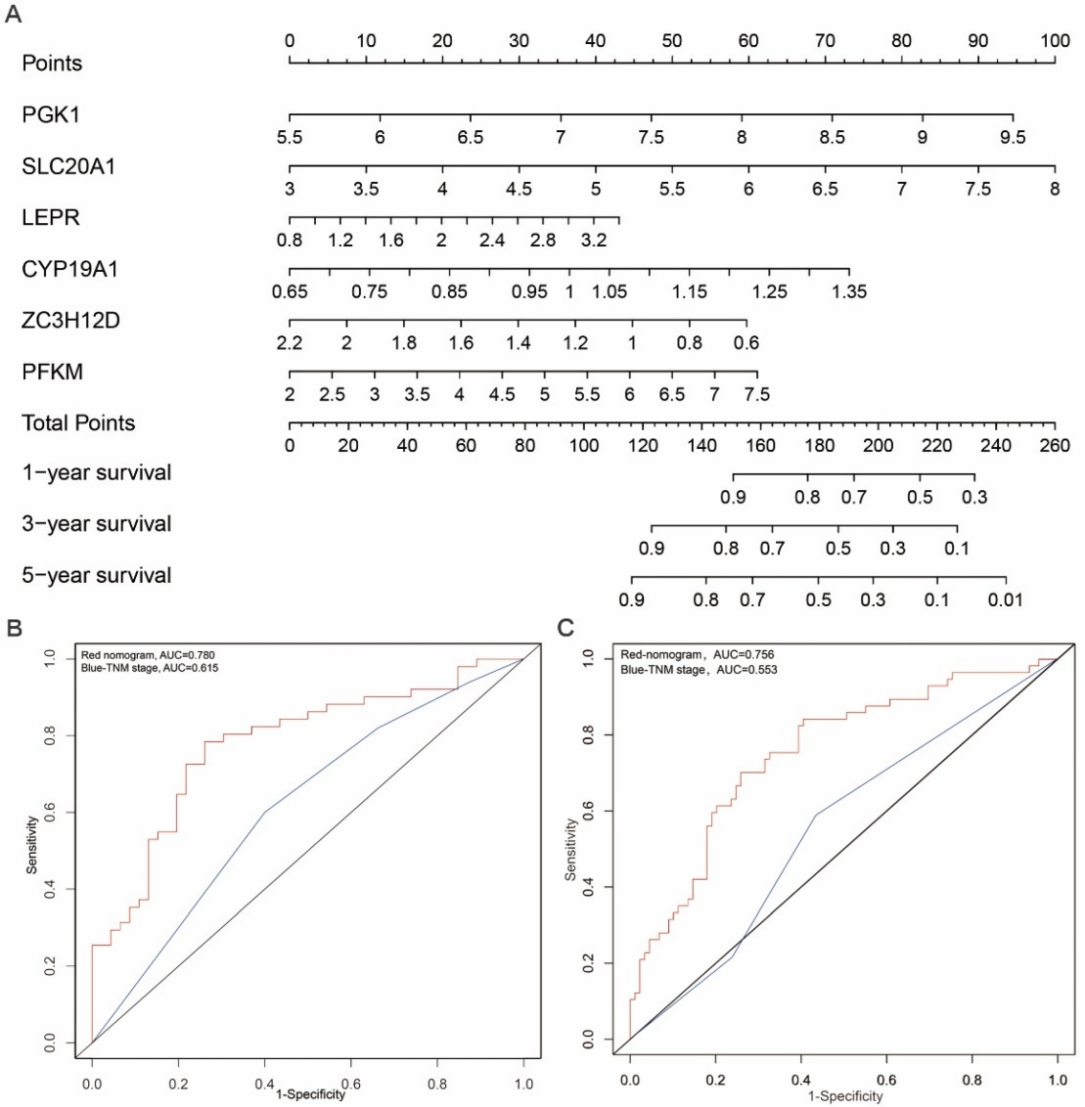
** Prognostic nomogram to predict the 1-,3-, and 5-year OS probabilities of tongue cancer patients.** (A) OS, overall survival; Nomogram was built based on prhRBPs' expression in TCGA train cohort. The expression level of each hub RBP times the coefficient value equal to a point. Total points equal to the sum of every single point. Based on the previous identified correlation between risk score and overall survival rate in TCGA cohort, the total points could be corresponded to a 1-,3-, and 5-year OS probability of tongue cancer patients. (B, C) Comparison of the predictive accuracy for 5-year OS between the nomogram and TNM stage. In the internal validation cohort (B), the AUC of the nomogram (0.780) was higher than the TNM stage (0.615). In the external validation cohort (C), the AUC of the nomogram (0.756) was higher than the TNM stage (0.553).

In order to develop a quantitative method for tongue prognosis, we integrated the six RBPs signature to establish a nomogram (Figure 6A). Based on the multivariate Cox analysis, points were assigned to individual variables by using the point scale in the nomogram. We draw a horizontal line to determine the point of each variable and calculate the total points for each patient by summing the points of all variables, and normalize it to a distribution of 0 to 100. We can calculate the estimated survival rates for tongue patients at 1, 3, and 5 years by drafting a vertical line between the total point axis and each prognosis axis, which might help relevant practitioners to develop clinical decision making for tongue patients. The predictive accuracy for the 5-year OS, as measured by AUC, was 0.780 in the TCGA internal validation cohort (Figure 6B). The nomogram was externally validated by an independent validation cohort of 97 tongue cancer patients from GEO database, and the AUC of the nomogram for predicting the 5-year OS was 0.756 (Figure 6C). The results demonstrated that the nomogram has good prognostic discrimination ability. In addition, as shown in Figure 6B and 6C, the nomogram displayed higher accuracy for predicting survival in both cohorts than the TNM staging system. Briefly, the AUCs of nomogram in both TCGA and GEO-validation cohort (0.780; 0.756) were higher than the TNM stage (0.615; 0.553). These results suggested that our nomogram is more accurate and useful for predicting OS of tongue cancer patients compared to the conventional TNM stage.

### Prognosis-related hub RBPs regulate cancer metabolic processes

**Figure 7:**
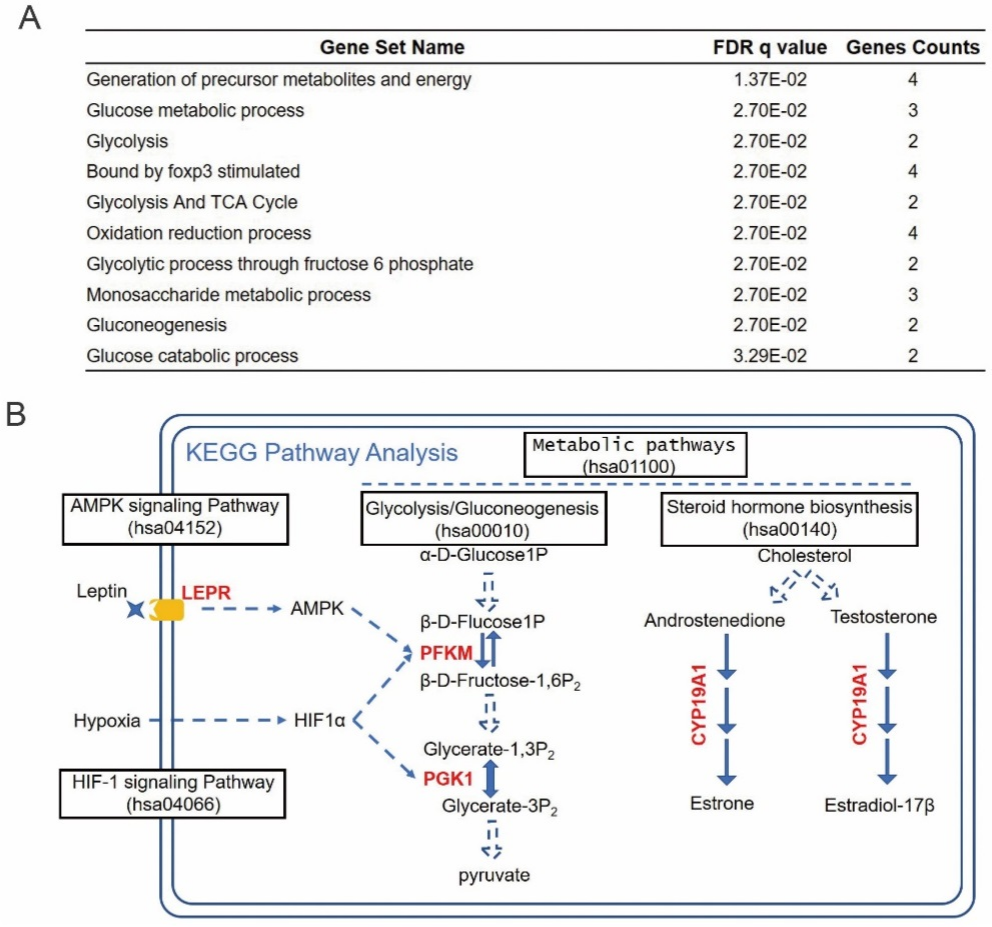
** Enrichment of prhRBPs in metabolic processes.** (A) GSEA analysis of prhRBPs. (B) KEGG pathway analysis of prhRBPs.

To investigate the functional mechanisms of the identified prognosis-related hub RBPs, we uploaded them to the online tool GSEA (https://www.gsea-msigdb.org/gsea/index.jsp) for functional enrichment analysis. The results strongly enriched the prognosis hub RBPs into metabolic processes, including generation of precursor metabolites and energy, glucose metabolic process, glycolysis and TCA cycle, etc (Figure 7A). The cancer-associated metabolic alterations have profound effects on gene expression, cellular differentiation and the tumor microenvironment 33. And it has become evident that the alteration of cancer metabolism has been considered as an emerging hallmark of tumorigenesis 33. Thus, our results indicated that these prognosis-hub RBPs might affect tongue cancer progression through regulating various metabolic processes. In order to determine the detailed role of the prognosis hub RBPs, we further uploaded these prognosis-related hub RBP genes to the online tool KEGG pathway analysis (https://www.genome.jp/kegg/tool/map_pathway2.html). KEGG analysis respectively enriched LEPR, PFKM, PGK1, and CYP19A1 in AMPK signaling pathway, HIF-1 signaling pathway, Glycolysis/Gluconeogenesis and steroid hormone biosynthesis (Figure 7B). AMPK, HIF-1 signaling pathways and Glucose metabolism are widely recognized and heavily pursued for treatment of metabolic diseases, such as a variety of cancers 34-37. Previous reports have demonstrated that the increased hormones occurred as cause of tongue cancer progression and revealed the components of the steroid biosynthetic pathways can be considered to be cancer biomarkers 38-41. Thus, the enrichment of LEPR, PFKM, PGK1, and CYP19A1 in the aforementioned pathways suggests these prhRBPs as potential therapeutic targets.

## Discussion

The abnormal expression of RBPs have been reported in various malignant tumor, and their expression correlates with patient prognosis 14-23. However, RBPs have not been thoroughly and systematically studied in tongue cancer. Global analysis of the functional impact of RBPs will provide new insights into tongue tumor therapy. In the present study, we identified 962 differently expressed RBPs between tumor and normal tissues based on TCGA retrieved tongue datasets. We systematically analyzed relevant biological pathways of these RBPs. By co-considering the WGNA results, we obtained tongue cancer highly correlated hub RBPs from the constructed co-expression network and PPI network. Moreover, we also performed univariate cox regression analysis, survival analyses, multiple stepwise cox regression analysis, and ROC analyses of the hub RBPs to further explore their biological functions and clinical significance. In addition, we constructed a risk model to predict tongue cancer prognosis based on the prognostic hub RBP genes. These findings may shed new light on developing novel biomarkers for the diagnosis and prognosis of patients with tongue cancer.

The aforementioned hub RBPs were selected by univariate cox regression analysis, survival analyses, and multiple cox regression analysis. A total of six hub RBPs were identified as prhRBPs, including LEPR, PFKM, PGK1, CYP19A1, SLC20A1, and ZC3H12D. Previous studies have reported that the expression of LEPR 42, PFKM 43, 44, PGK1 45, 46, CYP19A1 47, 48, SLC20A1 49, and ZC3H12D 50 were associated with tumorigenesis and progression in various tumor types, which support the further identification and exploring in tongue cancer. Next, we produced a risk model based on these hub RBPs, trained using TCGA cohort and validated by GEO retrieved tongue cancer patients. The ROC analysis revealed that these hub RBP genes signature with the better diagnostic capability to select out the tongue cancer patients with poor prognosis. Subsequently, a nomogram was built to predict 1, 3, and 5 years OS more intuitively. We also used the Kaplan Meier-plotter to detect the prognostic value of the hub RBPs genes, the results were basically consistent with the prognostic analysis results of TCGA cohort. These results suggested that the prognostic model of these hub RBPs genes have a certain value in adjusting treatment plans of tongue cancer patients.

Here, by performing the pathway enrichment analysis, we revealed that four of six hub RBPs (LEPR, PFKM, PGK1, and CYP19A1) are greatly enriched in metabolic processes. Cellular metabolism lies at the foundation of all biological activities. Unlike normal cells that are instructed to proliferate by extracellular signals, most cancer cells have acquired the ability to take up glucose cell-autonomously through the activation of oncogenes, including AMPK, HIF1α, and hence, acquire much more glucose for their oxidative metabolism 51. Here, we identified LEPR is the upstream signal of AMPK. The upregulation of AMPK and HIF1 can directly induce the expression of PFKM and PGK1. These two factors accelerate the use of glucose and thus helps the cancer cells to generate more substrate and energy it needs (Warburg effect). Deregulated glucose uptake has emerged as a hallmark of cancer metabolism 33. In our study, we did find the abnormally upregulation of LEPR, PFKM and PGK1 in tongue tumor patients and their upregulation are related to patients' poor overall survivals. Another metabolic pathway we identified was hormone biosynthesis, which the prognostic hub RBP CYP19A1 involved. CYP19A1 catalyzes the aromatization of androstenedione and testosterone to estrone and estradiol, respectively 52. Previous studies have suggested the potential involvement with sexual hormone receptors, and increased ER expression in tongue cancer 53, 54. Recent studies in revealing the positive relationship between ER and oral cancer also indicated the potential oncogenic role of CYP19A1 in tongue cancer 55, 56.

Although SLC20A1, also named PiT-1, is not annotated in KEGG database, a recent study also identified SLC20A1 as a marker of tumor cell metabolism 57. As one of the *SLC20* family members, SLC20A1 functioned as sodium-driven inorganic phosphate transporter, which consists of the basic tumor cell metabolite transporters among glucose, glutamine and inorganic phosphate 57. Several studies suggest that SLC20A1 are upregulated in tumor cells and have been considered to be important promoters of tumor progression 49. Beyond the Pi transport, SLC20A1 was also demonstrated for function in tumor cell proliferation and apoptosis 58. These reports not only indicated the potential role of SLC20A1 in the tongue cancer metabolism, but also supported the undiscovered oncogenic role SLC20A1 in tongue cancer progression.

ZC3H12D, also known as TFL or p34, is originally reported as a putative tumor suppressor in transformed follicular lymphoma and sporadic lung cancer 50, 59. Following work showed overexpression of ZC3H12D would significantly inhibitor TLR-induced JNK, ERK, and NF-κB 60. These findings suggest the suppressor role of ZC3H12D in tumorigenesis. In our study, we also found aberrant down-regulation of ZC3H12D in tongue cancer patients with better prognosis. Together, we identified the potential functional pathways of the prognosis-related hub RBPs.

RBPs are not only involved in all steps of RNA biogenesis, but also exhibit highly dynamic interactions with coding or non-coding RNAs to facilitate a variety of biological functions including cancer 9, 11, 61. For the prognosis-related hub RBPs identified in this work, several reports have shown the tumor-related functions directed by hub RBPs and their interacted non-coding RNAs in hepatocellular carcinoma (HCC), triple-negative breast cancer (TNBC), Prostate adenocarcinoma (PRAD) 62-64. It is reasonable to predict that the identified hub RBPs mediate the cancer related events through their associated RNAs in tongue cancer.

By using the six hub RBPs as variables, we built a nomogram applied for clinical prognostic evaluation. Our study had some advantages over previous reports. Previous studies applied nomogram based on all the types of oral cancer or head and neck cancer, instead of a specific type of cancer 65, 66, which may mask some cancer type specific phenomena due to the heterogeneity. Focusing on a specific type of cancer would produce a more accurate estimate of the risk causing patients' dying 67, 68. Here, we build a nomogram specifically applied for tongue cancer prognostic evaluation rather than oral cancer or head and neck cancer. Tongue cancer is a prevalent type in oral cavity with aggressive clinical behaviour and relatively low 5-year survival rate 1-4. Prognostic evaluation based on gene expression profile may also provide information about whether the patients will benefit from targeted molecular therapy 69. Collectively, compared to conventional TNM staging system, using the nomograms based on the identified hub RBPs may effectively improve the prediction of tongue cancer prognosis.

In summary, we systematically explored the expression and prognostic value of differently expressed RBPs by a series of bioinformatics analyses in tongue cancer. The prognostic risk model based on the hub RBP genes was constructed, and served as an independent prognostic factor for tongue cancer. In addition, we revealed these hub RBPs functioned through multiple critical tumor metabolic processes. Our results would contribute to show the pathogenesis of tongue cancer and to develop new treatment targets and prognostic molecular markers.

## Supplementary Material

Supplementary table S1.

## Abbreviations

RBP: RNA binding protein
DeRBP: differentially expressed RBPs
DceRBP: Differential co-expression RBP
prhRBP: prognosis-related hub RBP
OS: overall survival
ROC: receiver operating characteristic
AUC: area under the ROC curve
BP: biological process
CC: cellular component
MF: molecular function
